# Plasma Siglec-5 and CD163 as Novel Biomarkers for Fulminant Myocarditis

**DOI:** 10.3390/biomedicines10112941

**Published:** 2022-11-15

**Authors:** Yan Zhuang, Jin Wang, Huihui Li, Yanghui Chen, Chen Chen, Dao Wen Wang

**Affiliations:** Division of Cardiology and Hubei Key Laboratory of Genetics and Molecular Mechanisms of Cardiological Disorders, Tongji Hospital, Tongji Medical College, Huazhong University of Science and Technology, Wuhan 430074, China

**Keywords:** Siglec-5, CD163, biomarker, fulminant myocarditis

## Abstract

Fulminant myocarditis (FM) is the severest type of myocarditis and requires timely diagnosis and treatment. However, effective biomarkers for early diagnosis of FM are limited. First, 12 common inflammatory cytokines levels in the plasma of patients with FM were measured using human cytokine 12-Plex assay. Then, enzyme-linked immunosorbent assay (ELISA) was used to detect the plasma levels of another eight cytokines that we previously reported on. Moreover, a Spearman correlation test was employed to investigate the correlations between the plasma cytokine levels and the clinical parameters of patients with FM. Finally, receiver operating characteristic (ROC) curve analyses were performed to assess the diagnostic performance of plasma cytokine levels for the detection of FM. Five of the twelve common inflammation cytokines were significantly altered in patients with FM, but none of them was correlated with the severity of FM. Six of the eight significantly changed cytokines that we previously reported on were validated by ELISA. Among these, sST2, Siglec-5, and CD163 were negatively correlated with ejection fraction values. Furthermore, plasma Siglec-5 and CD163 levels were found to be associated with the severity of FM. Finally, both plasma Siglec-5 and CD163 showed outstanding diagnostic performance for FM. The current study identified plasma Siglec-5 and CD163 as valuable novel biomarkers for the early diagnosis of FM.

## 1. Introduction

Myocarditis is a myocardial inflammatory disease usually caused by infection (such as a virus), autoimmune disorders (such as sarcoidosis), poisoning (such as heavy metals), intoxication from drugs (such as anthracyclines), or abuse substances (such as cocaine) [1,2]. There are many different clinical signs and symptoms associated with it, from slight squeezing chest depression after activity, to acute left heart failure, cardiogenic shock, and even sudden death [3,4]. Acute myocarditis is frequently considered to be a mild and self-limited disease. The severest form of myocarditis, fulminant myocarditis (FM), is distinguished by fast onset, rapid progression, hemodynamic dysfunction, such as pump failure and circulation failure, which can develop very quickly, and simultaneous respiratory, liver, or renal failure as well. In the early stages of FM, patients may have a high fatality rate [4,5,6]. Therefore, early accurate diagnosis is critical for the treatment of patients with FM [7].

The gold standard for myocarditis diagnosis relies on endomyocardial biopsy, which is not routinely performed and whose sensitivity is limited [8,9]. Currently, the diagnosis of myocarditis tends to be established after confirming the presence of Lake Louise criteria via cardiac magnetic resonance imaging (MRI) [10]. However, cardiac MRI is not readily available under emergent circumstances. Both endomyocardial biopsy and cardiac MRI have limitations, especially their time-consuming nature.

Therefore, reliable and accessible diagnostic tools for the early diagnosis of FM constitute an urgent clinical need. Considering that FM is a severe inflammatory disease of the heart, and that the cytokine storm plays a central role in the pathophysiology of FM, we speculated that plasma-inflammation-associated cytokines may serve as potential biomarkers for FM [11]. Using human cytokines array analysis, our previous study found eight cytokines were altered significantly (seven increased and one decreased) in patients with FM at admission and recovered significantly at discharge; among these sST2 was a highly specific and sensitive biomarker for FM [12]. However, a cytokine storm is often associated with alterations of circulating levels of multiple cytokines and potential associations of other cytokines with FM, and whether these cytokines can be used as diagnostic markers for FM remain unclear.

In this study, based on human cytokines array data, we screened six cytokines which altered notably in the plasma of patients with FM at admission. After further analysis, we found potential correlations between the clinical parameters and the levels of Siglec5 and CD163 in the plasma of patients with FM, and determined the diagnostic performance of plasma Siglec-5 and CD163 for FM. Our study revealed novel associations between FM and plasma Siglec-5 and CD163, and identified plasma Siglec-5 and CD163 as novel specific and sensitive biomarkers for FM.

## 2. Methods

### 2.1. Study Population

All patients and control individuals were recruited at Tongji Hospital, Wuhan, China, between April 2017 and March 2021. The study was authorized by the Tongji Hospital and Tongji Medical College ethical review board (ID: TJ- C20160202), and it complied with the Declaration of Helsinki’s standards. Written consents were obtained from all participants after being fully informed. FM was diagnosed according to the 2009 International Consensus Group on Cardiovascular Magnetic Resonance in Myocarditis statement, the position statements from the 2013 European Society of Cardiology, and the 2017 Chinese Society of Cardiology expert consensus statement [13,14,15].

The following were the diagnostic criteria for FM: (1) the duration of acute HF symptoms was less than two weeks; (2) inotropic support or mechanical circulatory support resulting from hemodynamic instability was administered; (3) cardiac magnetic resonance imaging demonstrated the presence of myocarditis; (4) myocarditis was confirmed by endomyocardial biopsy before discharge. To exclude coronary disease, coronary angiography was utilized. The exclusion criteria were: (1) patients aged < 11 years; (2) patients who may have an acute coronary syndrome but have not undergone coronary angiography to differentiate it from FM; (3) patients with myocardial injury caused by sepsis; (4) patients with hypovolemic shock or unstable hemodynamics [14].

For human cytokine 12-Plex assay detection, 68 patients with FM and 27 healthy controls were enrolled.

For the further analysis and validation of data obtained from array analysis, we enrolled another 32 patients with FM and 16 controls as an independent cohort.

### 2.2. Human Plasma of 12 Inflammatory Cytokines Assay

The plasma levels of 12 common inflammatory cytokines in 68 patients with FM and 27 healthy controls were detected using an ABplex human cytokine 12-Plex assay kit (Cat#: RK04296, ABclonal Technology, Wuhan, China). In brief, covalently cross-linked antibody molecules or gene probes were utilized for different cytokines in specific coded microspheres; each coded microsphere corresponds to an analogous detection item. Firstly, the fluorescent coded microspheres for different test substances are mixed, and then the test substance or the amplified fragments to be tested are added, and the resulting complex reacts with the labeled fluorescein. Then, driven by the flowing sheath fluid, the microspheres pass through the red and green lasers in a single row. Finally, the red laser is used to determine the fluorescence code of the microspheres, and the green laser is used to determine the fluorescence intensity of the reporting molecules in the microspheres.

### 2.3. Enzyme-Linked Immunosorbent Assay

The plasma levels of 8 cytokines screened by human cytokine arrays analysis were measured using a standard enzyme-linked immunosorbent assay kit (Cat#: DST200, DSE100, DC1630, HSCT40, R&D System; Cat#: BMS297-2TEN, EHIL17BX5, BMS225-2, Invitrogen, Carlsbad, CA, USA; Cat#: RAB0433, Sigma-Aldrich, Saint Louis, MO, USA). We performed measurements according to the manufacturer’s instructions. For Siglec-5, the inter-assay CV value is less than 12% and intra-assay CV value is less than 10% (https://www.sigmaaldrich.cn/CN/zh/product/sigma/rab0433; accessed on 9 November 2022). For CD163, the inter-assay CV value is less than 6.7% and intra-assay CV value is less than 3.8% (https://www.rndsystems.com/cn/products/human-cd163-quantikine-elisa-kit_dc1630; accessed on 9 November 2022).

### 2.4. Statistical Analysis

We first evaluated the normality of the distributions of continuous data using the Kolmogorov–Smirnov test. Continuous values are shown as mean ± SD if normally distributed, or medians and first to third quartile (quartile 1–quartile 3) if not normally distributed. If distributions were normal, we used the unpaired Student’s *t*-test for comparisons between two groups. If distributions were non-normal, we used the Mann–Whitney test for the analysis of comparisons between two groups. Correlation was analyzed using the Spearman correlation test. We performed the analysis of the receiver operating characteristic (ROC) curves to evaluate the diagnostic performance of Siglec-5 and CD163. The optimal cut-off values of Siglec-5 and CD163 were established based on the highest Youden index as a summation of maximum sensitivity and specificity [16,17]. Patients were given a value of half the lower limit of detection if their cTnl or NT-proBNP concentrations were below the lower limit of detection. Patients were given a value of the upper detection limit if their cTnl values were above it. All diagrams were drawn using Prism 8 software (GraphPad Software, San Diego, CA, USA), SPSS 22 (IBM, Armonk, NY, USA), or R software (The R Foundation, Vienna, Austria). *p* values of <0.05 were considered to be significant.

## 3. Results

### 3.1. Plasma Levels of 12 Common Inflammatory Cytokines in Patients with FM

**Figure 1:**
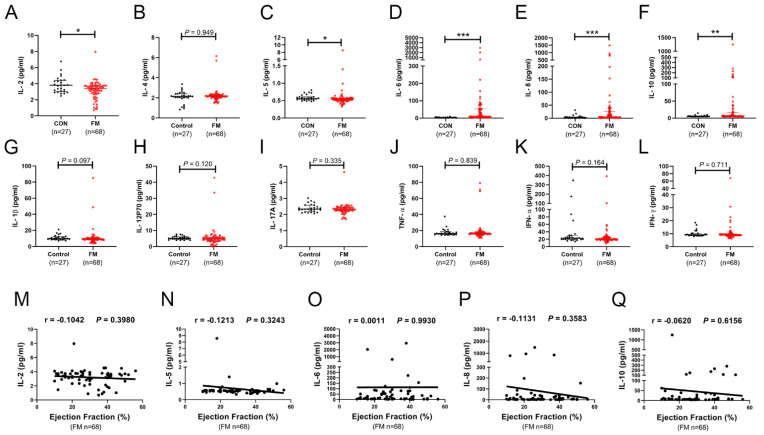
Plasma levels and correlation analysis of 12 common inflammatory cytokines in patients with fulminant myocarditis (FM). The plasma levels of IL-2 (**A**), IL-4 (**B**), IL-5 (**C**), IL-6 (**D**), IL-8 (**E**), IL-10 (**F**), IL-1β (**G**), IL-12P70 (**H**), IL-17A (**I**), TNF-α (**J**), IFN-α (**K**), and IFN-γ (**L**) in patients with FM (n = 68) and healthy controls (n = 27) were detected using an ABplex human cytokine 12-Plex assay kit. Data are presented as medians and quartile 1 to quartile 3 [Q1–Q3], and a Mann–Whitney test was used to elevate the differences; * *p* < 0.05, ** *p* < 0.01, *** *p* < 0.001. Correlation analysis of plasma IL-2 (**M**), IL-5 (**N**), IL-6 (**O**), IL-8 (**P**), and IL-10 (**Q**) with ejection fraction values in 68 patients with FM. r indicates the correlation coefficient.

To identify the cytokines associated with FM as diagnostic biomarkers, we firstly detected circulating levels of 12 common inflammatory cytokines in 68 patients with FM and 27 healthy controls using an ABplex human cytokine 12-Plex assay kit. The baseline clinical characteristics are presented in Table 1. The results show that only five of the twelve common cytokines were altered significantly (three increased and two decreased) (Figure 1A–L).

Then, we performed a correlation analysis to investigate whether these five common inflammatory cytokines were associated with the severity of FM. However, none of these five cytokines showed correlation with the ejection fraction values (a vital indicator of cardiac systolic function) of patients with FM (Figure 1M–Q).

These results suggest that these common inflammatory cytokines might not be appropriate candidates for diagnostic markers for FM.

### 3.2. Plasma Levels of Eight Cytokines Screened by Human Cytokine Arrays Analysis in Patients with FM

**Figure 2:**
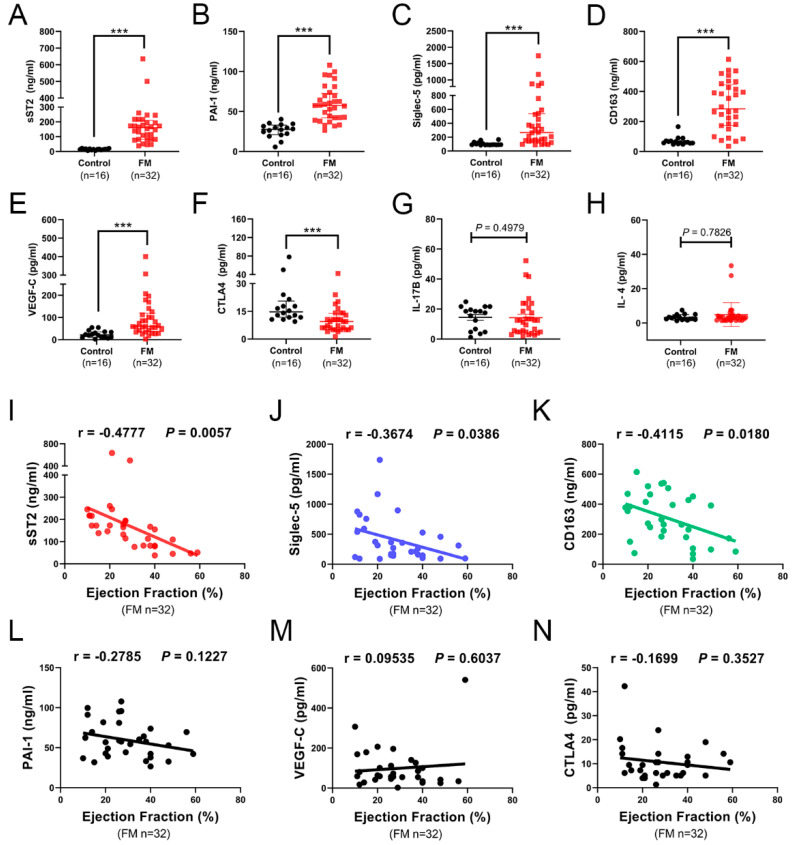
Plasma levels and correlation analysis of eight cytokines screened using human cytokine arrays analysis in patients with FM. Circulating concentrations of sST2 (**A**), PAI-1 (**B**), Siglec-5 (**C**), CD163 (**D**), VEGF-C (**E**), CTLA-4 (**F**), IL-17B (**G**), and IL-4 (**H**) in FM patients (n = 32) and control individuals (n = 16) were measured using ELISA. Data are presented as medians and quartile 1 to quartile 3 [Q1–Q3], and a Mann–Whitney test was used to elevate the differences; *** *p* < 0.001. Correlation analysis of plasma sST2 (**I**), Siglec-5 (**J**), CD163 (**K**), PAI-1 (**L**), VEGF-C (**M**), and CTLA-4 (**N**) with ejection fraction values in 32 patients with FM. r indicates the correlation coefficient.

To identify more sensitive and specific plasma inflammatory cytokines that are associated with the severity of FM as diagnostic biomarkers, we further analyzed and validated the alterations of eight cytokines which derived from the human cytokine arrays analysis in our previous study [12]. Here, 32 patients with FM and 16 controls were enrolled in another independent cohort (Table 1). All patients were confirmed as lymphocytic myocarditis using endomyocardial biopsy. We measured these eight cytokines levels in the plasma using enzyme-linked immunosorbent assay (ELISA). We found that six of them were significantly altered in the plasma of patients with FM at admission, while another two cytokines showed the same trend as the array analysis (Figure 2A–H).

To explore whether there were associations between these six inflammatory cytokines with ejection fraction values in patients with FM, we next performed a correlation analysis. Results showed that the plasma concentration of sST2 was negatively correlated with the ejection fraction values (Figure 2I), which was consistent with our previous study. In addition, we found that the plasma levels of Siglec-5 and CD163 were also negatively correlated with the ejection fraction values (Figure 2J–K). In contrast, plasma levels of PAI-1, VEGF-C, and CTLA4 were not correlated with the ejection fraction values (Figure 2L–N).

Our previous study indicated that sST2 was closely associated with FM. However, the cytokine storm occurring in FM is generally associated with the alterations of multiple cytokines, which suggests that other cytokines except for sST2 may also play an indispensable role in FM. Therefore, we further explored whether Siglec-5 and CD163 were also potential diagnostic biomarkers for FM.

### 3.3. Correlation between Plasma Siglec-5 and CD163 Concentrations with Fulminant Myocarditis-Related Clinical Parameters

**Figure 3:**
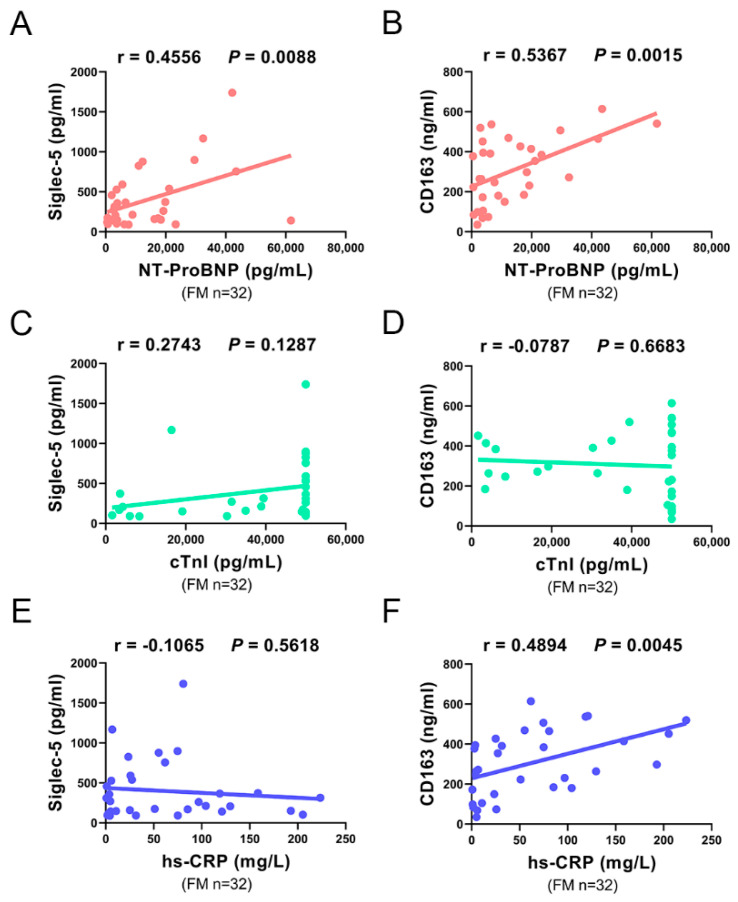
Correlation between plasma Siglec-5 and CD163 concentrations with fulminant myocarditis-related clinical parameters. Correlation analysis of plasma Siglce-5 and CD163 concentrations with N-terminal pro-B-type natriuretic peptide (NT-ProBNP) (**A**,**B**), cardiac troponin I (cTnI) (**C**,**D**), and high- sensitivity C- reactive protein (hs-CRP) (**E**,**F**) in 32 patients with FM. r indicates the correlation coefficient.

To further investigate the association of Siglec-5 and CD163 with FM, we performed a correlation analysis between plasma Siglec-5 and CD163 concentrations with fulminant myocarditis-related clinical parameters. We found that both plasma levels of Siglec-5 and CD163 were positively correlated with plasma NT-proBNP level, but not correlated with plasma cTnI level (Figure 3A–D).

In addition, the plasma CD163 level was obviously positively correlated with the concentrations of high-sensitivity C-reactive protein (hs-CRP) (an indicator of inflammation), suggesting that plasma CD163 might be associated with the systemic inflammatory response of patients with FM (Figure 3F). However, we did not find any association between the plasma level of Siglec-5 and the concentrations of hs-CRP (Figure 3E).

Collectively, these results indicate that the plasma levels of Siglec-5 and CD163 were associated with NT-proBNP, suggesting that they might be used as clinical indicators for the severity of FM.

### 3.4. Diagnostic Performance of Plasma Siglec-5 and CD163 Levels for the Detection of FM

**Figure 4:**
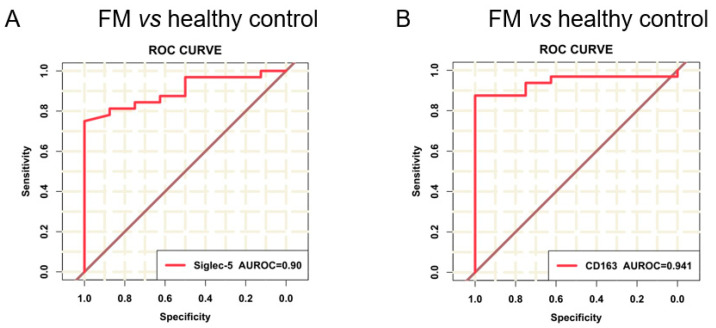
Diagnostic performance evaluation of plasma Siglec-5 and CD163 for FM using the ROC curve analysis. Receiver operating characteristic (ROC) curves of plasma Siglec-5 (**A**) and CD163 (**B**) in 32 patients with FM and 16 control individuals. AUROC indicates the area under the receiver operating characteristic.

To determine the diagnostic ability of plasma Siglec-5 and CD163 for FM, we performed an ROC analysis. As shown in Figure 4A,B, the ROC curves analysis revealed that plasma Siglec-5 and CD163 levels at admission could distinguish FM patients from control individuals with a good diagnostic performance (area under the curve (AUC) of 0.90 (0.82–0.98) for Siglec-5; AUC of 0.94 (0.87–1.00) for CD163). The optimal cutoff value of Siglec-5 for distinguishing FM patients from healthy individuals was determined to be 133.80 pg/mL, as well as 89.56 ng/mL for CD163, where the highest Youden index was achieved with the maximal summation of sensitivity and specificity (Table 2). At these thresholds, both Siglec-5 and CD163 showed high sensitivity and specificity (the sensitivity of 81.3% and specificity of 87.5% for Siglec-5; the sensitivity of 87.5% and specificity of 87.5% for CD163).

These results indicate that the ability of plasma Siglec-5 and CD163 levels to distinguish FM from control individuals was excellent.

## 4. Discussion

In this study, we identified plasma Siglec-5 and CD163 as novel valuable biomarkers of fulminant myocarditis. We found novel correlations between plasma levels of Siglec-5 and CD163 and the severity of FM, and the diagnostic ability of plasma Siglec-5 and CD163 to discriminate patients with FM from control individuals was confirmed using ROC analysis.

In clinical settings, the diagnosis of FM remains a challenge because of the lack of easily accessible diagnostic methods that are both sensitive and specific [13]. To identify some sensitive plasma inflammatory cytokines that are associated with FM as diagnostic biomarkers, we firstly detected 12 common inflammatory cytokines levels in the plasma of 68 patients with FM and 27 healthy controls. Only five common inflammatory cytokines showed significant alteration in patients with FM, but none of them was correlated with the ejection fraction values of FM patients. These results suggested that these common cytokines were not appropriate candidates for diagnostic markers for FM.

Then, we further investigated the eight cytokines that we previously screened by human cytokine arrays analysis, and found that six of them were significantly altered in the plasma of patients with FM, among which sST2, Siglec-5, CD163 were negatively correlated with the ejection fraction values. Considering that other cytokines except for sST2 might also be important in the cytokine storm occurring in FM, we further explored the potential association of plasma Siglec-5 and CD163 levels with the severity of FM, and determined the value of plasma Siglec-5 and CD163 as novel biomarkers for FM.

Our results revealed that both plasma levels of Siglec-5 and CD163 were positively correlated with the plasma NT-proBNP level, a well-established indicator for cardiac dysfunction. However, we found that there was no correlation between the plasma levels of Siglec-5 and CD163 with the plasma cTnI level. The primary reason might be the significantly increased cTnl concentrations above the upper detection limit that were assigned a value of the upper detection limit (50,000 pg/mL). Interestingly, we found the plasma CD163 level was obviously positively correlated with the plasma hs-CRP level, a commonly used biomarker of systemic inflammation. Thus, the plasma CD163 level might also be an indicator for the systemic inflammation caused by FM.

The Siglec family of proteins are sialic-acid-binding immunoglobulin-like lectins that facilitate cell–cell interactions and adjust cellular activities in the innate and adaptive immune systems by recognizing glycans [18,19]. The macrophage receptor sialoadhesin/Siglec-1 was the first Siglec to be thoroughly described [20]. Siglec-5 is a member of the CD33-related Siglec subfamily, expressed in monocytes, neutrophils, and macrophages. It has four external Ig-like domains and two intracellular tyrosine-based signaling motifs [21,22]. There have been numerous reports of the alteration of soluble Siglec-5 during diseases, including patients with primary Sjogren’s syndrome (pSS) having higher levels of Siglec-5 in their saliva than controls, and type 2 diabetes mellitus (T2DM) patients with critical limb ischemia (CLI) having significantly higher plasma levels of Siglec-5 than those without CLI [23,24]. Since Siglec-5 is predominantly expressed on the surface of immune cells, elevated Siglec-5 in these diseases may indicate the alteration of the immune cells ratio and the dysregulation of the immune system, which is similar to FM [11].

CD163 is a 130-kDa transmembrane protein that is a member of the cysteine-rich scavenger receptor superfamily type B, which was originally described as a scavenger receptor for hemoglobin–haptoglobin complexes. It is primarily expressed in M2c macrophages that infiltrate into tissues during the “healing phase” of inflammation [25,26]. Many inflammatory disorders were associated with the elevated plasma levels of CD163, such as hemophagocytosis and the associated macrophage activation syndrome [27,28,29]. The systemic inflammatory response syndrome caused by these disorders usually poses aberrant lymphohistiocytic activation with a complicated and mostly unidentified origin [30]. Our array also indicated an inflammatory cytokine storm at the onset of FM [12]. Therefore, the increased plasma levels of CD163 in patients with FM might be correlated with the imbalance of the immune system and the disturbance of systemic inflammatory responses in FM patients.

This study had some limitations. To our knowledge, this study provides the first evidence that plasma Siglec-5 and CD163 are associated with the severity of FM and could be novel biomarkers for FM in the acute phase. Multiple center prospective trials are needed to validate these biomarkers. Moreover, the role of Siglec-5 and CD163 in the pathophysiological mechanism of FM remains to be addressed in future studies.

Taken together, plasma Siglec-5 and CD163 could be sensitive and specific inflammation-associated biomarkers for FM, which might provide relatively fast and accessible diagnosis for FM.

## Figures and Tables

**Table 1:** Baseline clinical characteristics of human cytokine 12-Plex assay detection cohort and human cytokine arrays analysis validation cohort.

	Human Cytokine 12-Plex Assay Detection Cohort		Human Cytokine Arrays Analysis Validation Cohort	
	Control (n = 27)	FM (n = 68)	Control (n = 16)	FM (n = 32)
Age (years)	36.2 ± 10.0	36.1 ± 13.5	33.2 ± 11.4	37.2 ± 14.7
Male/Female (n/n)	15/12	37/31	9/7	16/16
LVED (cm)	4.8 (4.6–5.0)	4.7 (4.4–5.3)	4.7 ± 0.7	4.8 ± 0.3
LVEF (%)	62.0 (59.0–66.0)	30.0 (22.0–41.0) *	62.4 ± 4.1	29.5 ± 13.1 *
NT-proBNP (pg/mL)	<70.0	7745.0 (3168.0–18,374.0) *	<70.0	7126.5 (3427.0–19,538.5) *
cTnI (pg/mL)	<1.9	38,746.2 (16,446.1–50,000.0) *	<1.9	50,000.0 (24,737.1–50,000.0) *

FM, fulminant myocarditis; LVED, left ventricular end-diastolic volume; LVEF, left ventricular ejection fraction; NT-proBNP, N-terminal pro-brain natriuretic peptide; cTnI, cardiac troponin I. Data are presented as mean ± standard deviation (SD) if normally distributed, or medians and first to third quartile (Q1–Q3) if not normally distributed; * *p* < 0.05 vs. control (Student’s *t*-tests and Mann–Whitney tests were used to calculate the significance).

**Table 2:** Diagnostic performance of Siglec-5 and CD163 to distinguish FM from control individuals.

	AUC (95% CI)	Cut-Off Value	Sensitivity (%)	Specificity (%)	Accuracy (%)	PLR	NLR	PPV (%)	NPV (%)
**Siglec-5**	0.90 (0.82–0.98)	133.80 pg/mL	81.3	87.5	83.3	6.504	0.214	92.9	70.0
**CD163**	0.94 (0.87–1.00)	89.56 ng/mL	87.5	87.5	87.5	7.000	0.143	93.3	77.8

AUC, area under the curve; PLR, positive likelihood ratio; NLR, negative likelihood ratio; PPV, positive predictive value; NPV, negative predictive value.

## Data Availability

The authors declare that all data supporting the findings of this study are contained within the article file.
